# Editorial: Perspective-Taking, Self-Awareness and Social Cognition in Neurodegenerative Disorders, Cerebral Abnormalities and Acquired Brain Injuries (ABI): A Neurocognitive Approach

**DOI:** 10.3389/fpsyg.2020.614609

**Published:** 2020-11-30

**Authors:** Sara Palermo, Antonella Carassa, Rosalba Morese

**Affiliations:** ^1^Department of Neuroscience, Center for the Study of Movement Disorders, University of Turin, Turin, Italy; ^2^European Innovation Partnership on Active and Healthy Ageing, Bruxelles, Belgium; ^3^Faculty of Communication, Culture and Society, Università della Svizzera Italiana, Lugano, Switzerland; ^4^Faculty of Biomedical Sciences, Institute of Public Health, Università della Svizzera Italiana, Lugano, Switzerland

**Keywords:** social cognition, perspective-taking, empathy, self-awareness, neurocognitive approach, neurodegenerative disorders, acquired brain injury

Human beings are not isolated entities but are embedded in a network of social relationships that influence them and by which they are influenced. A fundamental characteristic of the human being is the ability to cooperate, which has been a crucial step in the evolution of complex social interactions (Tomasello, 2009; Morese et al., 2016; Lo Gerfo et al., 2019).

According to Frith (2008), social cognition concerns the psychological processes that allow individuals to live in different social contexts, to benefit from belonging to social groups. It makes possible various aspects of daily life that are based on empathic processes, such as the perception and recognition of the emotions of the other and the consequent appropriate behavior. The discoveries of recent decades on the mirror neuron system offer an important contribution to the development of knowledge about the neurophysiological correlates underlying social cognition. This progress can be considered an important step in understanding people's actions, emotions, and beliefs.

This new trend has opened a new interdisciplinary field, called “social neuroscience,” dedicated to the understanding of the relationship between social psychology and neuroscience. The aim is to investigate the links between mind, body, and behavior by analyzing how social interactions affect cognitive abilities, brain, and physiological functioning (Cacioppo, 2002). This branch of neuroscience aims to analyze phenomena at three levels of analysis: the social level, which concerns the motivations and social factors that influence behavior; the cognitive level, as a mechanism of information processing; and the neural level, which concerns the brain mechanisms underlying the cognitive processes involved.

The Research Topic here presented aims to provide knowledge on the (neuro)psychological models and the neuro-functional architecture of social and affective processes to foster the understanding of the cognitive, emotional, and behavioral functioning of individuals with specific disorders. Some cognitive processes such as metacognition, perspective-taking, empathy, self-awareness, executive functions and social cognition are not just a matter of debate of neuroscience, but they have raised increasingly attention as they have clinical effects on patients' quality of life, compliance with treatment, and prognosis. Moreover, these cognitive higher-order cognitive processes are crucial for interpersonal relationships and effective communication. This is even more true in the case of clinical settings: deficits in these domains have a deleterious impact on the doctor-patient relationship and, subsequently, on therapeutic interventions. There is also more. The neurocognitive approach has formerly emphasized the association among brain pathology, metacognitive-executive dysfunctions, and self-awareness reduction.

The goal of this Research Topic is to bring together theoretical models and experimental research pertaining to all these aspects in neurodegenerative disorders, cerebral abnormalities, and acquired brain injuries. The intention is to provide the reader with the most up-to-date perspective on how the interplay between neurophysiological mechanisms and neuropsychological factors leads to multifaceted and highly organized behaviors.

This e-book comprises a special issue collection of 11 contributions represented by one full review, one general commentary, two perspective articles, one study protocol, three original research articles, one brief research report, and two case reports, which are classified into Part A: Neurodegenerative Disorders; Part B: Acquired Brain Injuries.

## Neurodegenerative Disorders

Anosognosia and reduced self-awareness are frequently used as alternative expressions and an overlay between them has been emphasized. This is a delicate matter since according to the interpretation given to those terms, evaluation and understanding of the phenomenon change (Morese et al., 2018). Interpretative models and vocabulary are discussed in an interesting commentary (Bertrand et al.). The authors explore fundamental issues such as executive anosognosia, apathy, and error Monitoring.

A reduction in self-awareness is a crucial aspect in the symptomatology of various neurodegenerative disorders. An intriguing theoretical proposal regarding the use of virtual reality as fruitful addition to self-awareness assessment is presented (Muratore et al.). ICT-IoT technology can also promote a perspective change about neurodegenerative diseases that encourages a more inclusive view of patients. To ensure this, Dementia-Friendly Communities have recently been developed (Morganti et al.). In this scenario, a personalized approach that enhances the emotional state of cognitively impaired patients seems to be increasingly necessary: the Lüscher color test is proposed as a simple and unconventional approach to understand the emotional life of Alzheimer's disease patients (Maserati et al.). “Unconventional” approaches and neuroimaging findings prove to be useful in pathologies with different etiopathogenesis especially when atypical. The report of a case of Creutzfeldt-Jakob disease with an unusual clinical presentation is presented here. The clinical picture was characterized by dynamic aphasia in the context of a prominent dysexecutive syndrome and a lack of emotional insight and concern over the health status (Prodi et al.).

The damage of metacognitive-executive functions has previously been associated also with dyskinesias-reduced-self-awareness (Palermo et al., 2017a, 2018a,b; Palermo et al., 2019b) and impulse control disorder (Palermo et al., 2017b; Palermo and Morese, 2018) in Parkinson's disease (PD). A perspective article on altruistic punishment and impulsivity in PD suggested that metacognitive-executive functions and neurophysiological abnormalities, which associates disinhibition with volition, are also associated with social cognition impairment (Morese and Palermo). This kind of symptomatology requires a person-centered perspective capable of addressing the unmet needs of PD patients (Palermo et al., 2019a). In line with the above, a PD-specific intervention for increasing patients' mindfulness—and thereby reducing impaired self-awareness for motor symptoms—has successfully been tested and described: IPSUM (Buchwitz et al.). Its impact on self-awareness and patients' daily living is now being assessed. Another original approach to the disease sheds light on a possible use of complex action observation to improve or slow the deterioration of motor abilities and levodopa-induced dyskinesias (Palermo et al.). Never had anyone tried to study the neural correlates involved in empathy and embodiment in PD through observation of choreutical arts.

The section closes with an interesting study on resting-state functional correlates (RS-FC) of social cognition in multiple sclerosis (Bisecco et al.). For the first time, an association between social cognition and RS-FC changes of DMN, executive and limbic networks has been verified.

## Acquired Brain Injuries

Traumatic brain injury (TBI) can be serious partly due to the challenges of assessing and treating its neurocognitive and affective sequelae. Impairment in social cognition and its neural underpinnings have not been explored thoroughly in TBI. Findings on the cognitive and affective consequences of TBI in relation to neuropsychological testing strategies, to neurobiological and neuroimaging correlates, and to patient age at and assessment time after injury are presented in a full review (Calvillo and Irimia).

A last research focuses on moral emotions, aiming to investigate the differences in moral functioning that characterize TBI patients. The Moral Emotional Agent (MEA) methodology and implications for the design of rehabilitation applications based on virtual agents are presented (Ceccaldi et al.).

## Author Contributions

All authors listed have made a substantial, direct and intellectual contribution to the work, and approved it for publication.

## Conflict of Interest

The authors declare that the research was conducted in the absence of any commercial or financial relationships that could be construed as a potential conflict of interest.
